# A Novel Low-Cost, Large Curvature Bend Sensor Based on a Bowden-Cable

**DOI:** 10.3390/s16070961

**Published:** 2016-06-24

**Authors:** Useok Jeong, Kyu-Jin Cho

**Affiliations:** School of Mechanical and Aerospace Engineering/IAMD, Seoul National University, Seoul 08826, Korea; snopyy@snu.ac.kr

**Keywords:** bend sensor, flexible sensor, shape sensor, bend measurement, low-cost sensor, Bowden-cable, cable conduit

## Abstract

Bend sensors have been developed based on conductive ink, optical fiber, and electronic textiles. Each type has advantages and disadvantages in terms of performance, ease of use, and cost. This study proposes a new and low-cost bend sensor that can measure a wide range of accumulated bend angles with large curvatures. This bend sensor utilizes a Bowden-cable, which consists of a coil sheath and an inner wire. Displacement changes of the Bowden-cable’s inner wire, when the shape of the sheath changes, have been considered to be a position error in previous studies. However, this study takes advantage of this position error to detect the bend angle of the sheath. The bend angle of the sensor can be calculated from the displacement measurement of the sensing wire using a Hall-effect sensor or a potentiometer. Simulations and experiments have shown that the accumulated bend angle of the sensor is linearly related to the sensor signal, with an R-square value up to 0.9969 and a root mean square error of 2% of the full sensing range. The proposed sensor is not affected by a bend curvature of up to 80.0 m^−1^, unlike previous bend sensors. The proposed sensor is expected to be useful for various applications, including motion capture devices, wearable robots, surgical devices, or generally any device that requires an affordable and low-cost bend sensor.

## 1. Introduction

Bend sensors have been widely used in various applications, from simple angle measurements to flexion and extension measurements of the body joints [1,2,3,4,5,6,7,8,9,10,11] and shape-sensing in mechanical structures [12,13,14]. Flex sensors and optical fiber sensors have proved useful in these applications.

A flex sensor is a thin film printed with conductive ink whose resistance changes depending on the bend curvature and angle of the sensor [1,15]. It is the most affordable bend sensor on the market, and several companies sell off-the-shelf versions of various dimensions [16,17]. Some research [12,13,14,18] and do-it-yourself projects [2] use flex sensors because they are cheap, easy to integrate, and do not require complicated signal processing. Motion capture gloves measuring the posture of the fingers using flex sensors have been developed [1,3,4,5] and commercialized [6] because they can be easily attached to fabric without adding volume. However, flex sensors tend to have large nonlinearities, including time-dependent creep and a nonlinear relationship between curvature and resistance change [3,4,19,20]. The nonlinearity of flex sensors prevents them from measuring large curvatures and the absolute angles of objects. They are also limited in length (maximum, 95.25 mm for the Spectra Symbol flex sensor) and so cannot measure a bend change for a large area [4].

Optical fiber-based sensors detect bending by measuring changes of intensity, phase, wavelength, or polarization of the light inside a bending optical fiber [3,4]. The elaborate and intensive techniques used to manufacture the optical fiber and sophisticated signal processing of the light spectrum enable highly sensitive measurements of fiber shape changes [21]. Also, these sensors take advantage of the physical properties of optical fibers, including small diameter and flexibility. Optical fiber-based sensors can be divided into two types depending on whether they measure the spectrum or the overall intensity of light [22]. Grating-based sensors including fiber Bragg gratings (FBGs) and long-period fiber gratings (LPFGs) measure the intensity variation of the fiber’s resonant dip wavelength [23,24,25,26]. An expensive device—an interrogator—is used to measure and analyze the spectrum of the light, and complicated signal processing is required to convert the spectrum data into shape information [3,4,21]. It is possible but difficult to minimize the effect of temperature and nonlinearities via special fabrication techniques [24,27]. Optical fiber-based sensors that measure light intensity were proposed [7,8,22] as a low-cost alternative to spectrum-analyzing sensors. These measure intensity changes in the light source of light-emitting diodes (LEDs) with photodiodes as the fiber bends. However, this type of sensor is used only to measure small curvatures [22].

Electronic textiles (e-textiles) are an emerging technology that can be used for bend sensing [9,10,11]. The fabric or thread used to make these textiles changes its electric properties depending on the stretch of the material. They are advantageous for wearable devices because the sensing material itself can be an integral part of the device. However, electronic textiles show high nonlinearities [11] and have limited application other than for wearable devices.

This paper proposes a novel bend sensor based on the Bowden-cable that operates on principles completely different from those at work in flex sensors and optical fiber-based sensors. A Bowden-cable is a wire-driven mechanism that consists of an inner wire and an outer sheath that transmits force through the inner wire. It provides high degrees of freedom in the routing path because the transmission consists of flexible components. It has been used in bicycle brake systems for decades and in many robotic applications, from exoskeleton robots [28,29,30,31] to surgical devices [32,33,34,35]. One issue with Bowden-cables is property changes [36], especially related to position error of the inner wire originating from the varying shape of the coil sheath [32,33,34,35].

**Figure 1:**
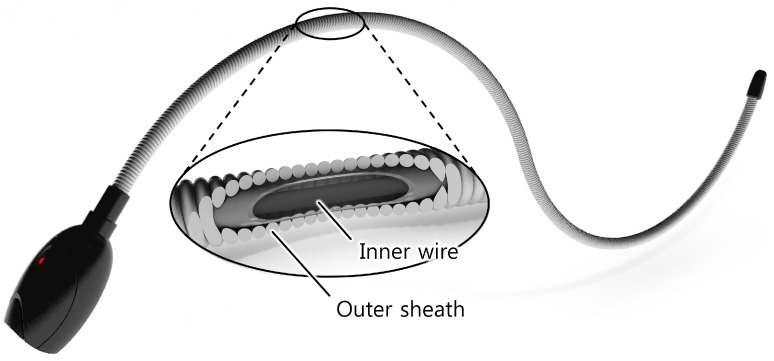
A Bowden-cable–based bend sensor consists of an inner wire and an outer sheath.

This study takes advantage of position error in the Bowden-cable to detect the bend angle of the sheath. When one end of the inner wire is fixed to the sheath, the displacement change of the inner wire can be measured on the other end of the sheath. There is a linear relationship between the displacement change of the inner wire and the accumulated bend angle of the sheath such that the sheath can be used as the sensor body of a bend sensor. The design and material of the proposed sensor is simple, low cost, and does not require any special fabrication techniques or complicated signal processing. Based on these attributes, we used a Bowden-cable to develop a low-cost, easy-to-use bend sensor (Figure 1) capable of sensing over a wide range of accumulative bend angle with a large curvature (up to 450° bend angle and 80.0 m^−1^ curvature for the first prototype).

This paper will discuss the basic concept, modeling, and preliminary evaluation of the proposed sensor. The concept and basic design of the sensor will be described in Section 2. The modeling of the proposed bend sensor’s bend angle, bend curvature, and sensor output will be discussed in Section 3. Characteristics and considerations of the proposed sensor will be covered in Section 4. Finally, Section 5 and Section 6 will describe development of a prototype and our preliminary experimental results for it.

## 2. Concept and Design

### 2.1. Principle

**Figure 2:**
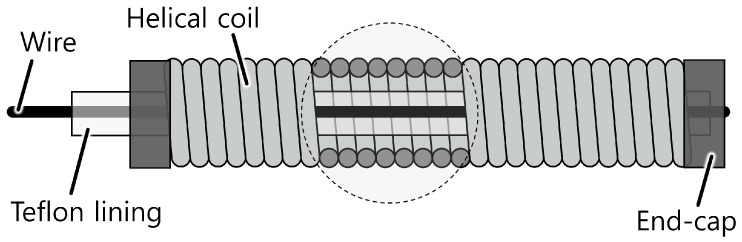
Structure of the bending part of the proposed bend sensor, which consists of a helical coil, an inner wire, a Teflon^®^ lining and an end-cap.

**Figure 3:**
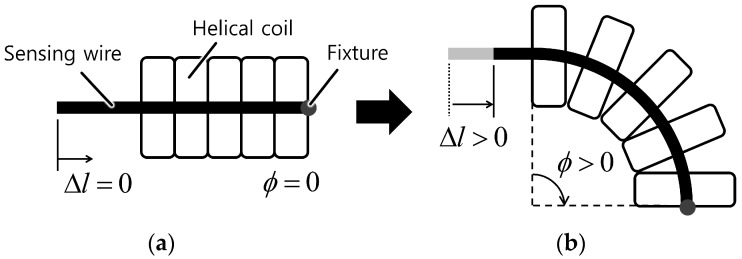
Basic working principle of the proposed bend sensor using a Bowden-cable. (**a**) When the sensor is straight; (**b**) When the sensor is bent.

The working principle of the proposed bend sensor starts from the idea that the displacement of the line passing through the center of a helical coil increases as the coil bends, as shown in Figure 2 and Figure 3. This occurs because each coil pitch remains in contact inside the curvature but the gap between each coil pitch on the outside of the curvature increases, as shown in Figure 3b. This results in a displacement change of the center line (this is linearly related to the bend angle of the helical coil, which will be addressed in more detail in the next section). The displacement change can be measured with a wire that is placed through the center of the coil and fixed at one end of the coil and a displacement sensor such as a potentiometer or a Hall-effect sensor.

**Figure 4:**
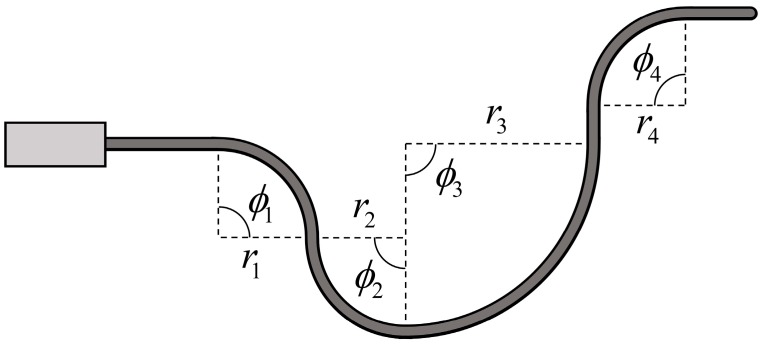
Sensor bending with multiple bending points. The displacement change of the center line is proportional to the accumulated bend angle throughout the helical coil, *φ* = *φ*_1_ + *φ*_2_ + *φ*_3_ + *φ*_4_.

This idea can be extended to the whole body of the sheath: Central displacement changes of the sensor are related to the accumulated bend angle of the sensor (depicted in Figure 4).

### 2.2. Maintaining Internal Tension

**Figure 5:**
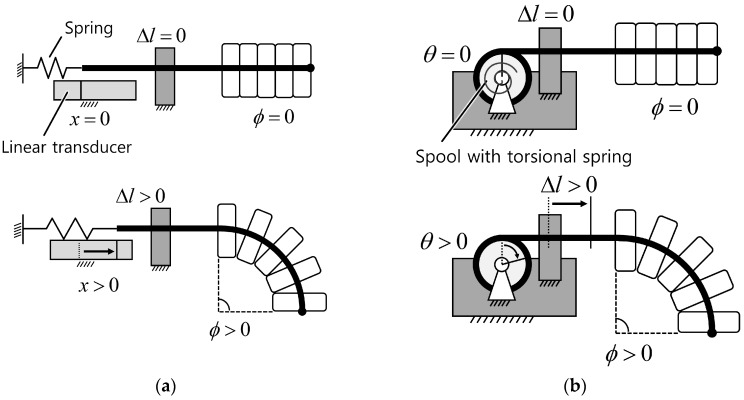
Two ways of maintaining internal tension and measuring the displacement change of the sensing wire. (**a**) Using a linear spring and a linear transducer; (**b**) Using a spool with a torsional spring and a rotational transducer.

The tension of the sensing wire inside the helical coil should be maintained over a certain range to prevent it from becoming slack, which would prevent accurate measurement of the central displacement change. There are two possible ways to keep the sensing wire taut. One is to use a passive element such as a spring to provide an elastic force to the sensing wire. Figure 5 shows examples of this method using a linear and a torsional spring. The free end of the sensing wire can be connected to a linear spring (Figure 5a) or wound around a spool with a torsional spring (Figure 5b). The spring should have some initial stretch to keep the sensing wire taut, even for a straight-shaped sensor. Inner tension increases as the sensor bends, and central displacement of the coil increases as well. In this case, the operating range of the spring should cover the travel range of the sensing wire, which is determined by the bend sensing range and the design parameters of the sensor. The other method is to maintain internal tension using an actuator such as an electric motor or piezo motor. This method can provide either constant or variable tension to the sensing wire, which enables the sensor to have variable bending stiffness or optimizes hysteresis properties. In all cases, tension should be maintained within a range that induces only negligible elongation of the wire. The wire stretch should be considered when calculating the bend angle if elongation of the wire is more than negligible.

### 2.3. Displacement Change Measurement

One end of the sensing wire is attached to the end of the helical coil, and the other free end of the sensing wire is connected to a displacement sensing device to measure the central displacement change of the helical coil. There are two possible ways to measure the position of the free end of the sensing wire. The first method merely measures the linear position of the free end of the sensing wire using a linear position transducer (Figure 5a). Available linear position transducers include potentiometers, linear variable differential transformers (LVDTs), and Hall-effect sensors. The second method measures the rotation angle of a spool wound by a sensing wire (Figure 5b). In both cases, the operating range and resolution of the displacement transducer influence the performance of the bend sensor. For example, it is preferable to choose a linear potentiometer that has a travel length as close as possible to the maximum range of the central displacement change of the sensor.

## 3. Modeling

This section provides a sensor model that describes the relationship between bend angle and curvature of the sensor, the displacement changes of the sensing wire, and sensor output. The resultant model shows that the bend angle of the sensor and the sensor output are linearly related within an allowable curvature range.

### 3.1. Basic Model

**Figure 6:**
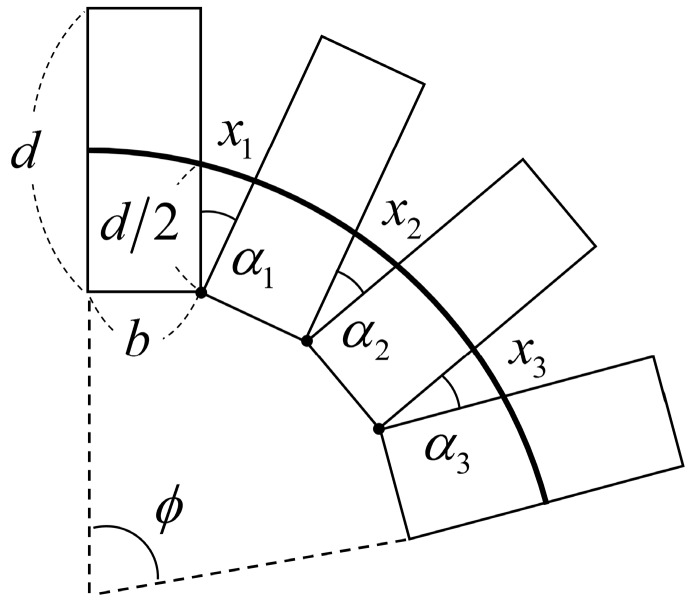
Schematic of a bent helical coil showing the displacement change of the line passing through the center of the coil. Each coil pitch uniformly bends by *α*, resulting in a central displacement increment of *x*.

Figure 6 delivers the basic modeling concept for the relationship between bend angle and the central displacement change of the coil. Each coil pitch is represented as a rectangle with coil wire diameter, *b*, and outer diameter, *d*. The vertexes of the rectangles on the inside of the bend keep in contact as the helical coil bends, and the angle between each coil pitch uniformly increases. Total bend angle, *φ*, and length of the sensor, *L*, can be represented by the number of coil pitches, *n*:

(1) $$\phi =\sum_{i=1}^{n}\alpha_{i}$$

(2) $$L=\sum_{i=1}^{n}b$$

The small change in the central displacement between two pitches is represented as *x* and can be calculated using a cosine rule:

(3) $$x=\frac{d}{2}\sqrt{2\left(1-\text{cos}\;\alpha \right)}=\frac{d}{2}\sqrt{2\left(1-\text{cos}\;\frac{b}{L}\phi \right)}$$

Then the central displacement changes of the helical coil along the sensor, ∆*l*, can be derived from the summation of the displacement changes:

(4) $$\Delta l=\sum_{i=1}^{n}x_{i}=\frac{dL}{2b}\sqrt{2\left\{1-\text{cos}\left(\frac{b}{L}\phi \right)\right\}}$$

**Figure 7:**
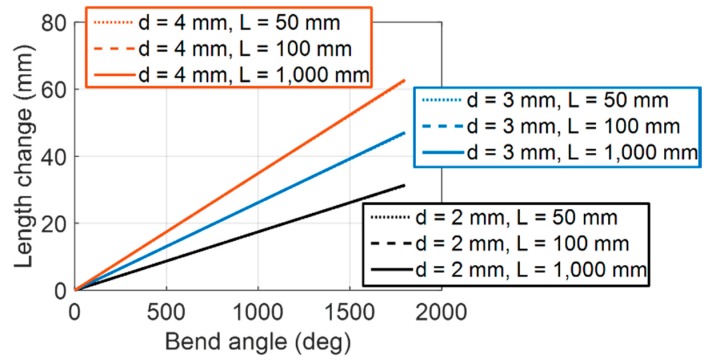
Simulation result of derived Equation (3) when coil wire diameter *b* = 0.5 mm. It shows the linear relation between bend angle and the central displacement change, although Equation (3) contains nonlinear functions. The curves with different length, *L*, are overlapped with the curves for the same coil diameter, *d*.

Figure 7 illustrates the simulation result of the derived Equation (3), showing the relationship between the central displacement change of the helical coil and the bend angle of the sensor. The simulation result indicates that it is possible to simplify the mathematical model.

### 3.2. Linearity

To further analyze the linearity of the bend angle and the central displacement change, Equation (3) can be expressed as the curvature of the sensor bend, *κ*:

(5) $$\kappa =\frac{\phi }{L}$$

(6) $$\Delta l=\frac{d}{2b\kappa }\sqrt{2\left\{1-\text{cos}\left(b\kappa \right)\right\}}\cdot \phi =A\left(\kappa \right)\cdot \phi$$

(7) $$A\left(\kappa \right)=\frac{d}{2}\cdot \frac{\sqrt{2\left\{1-\text{cos}\left(b\kappa \right)\right\}}}{b\kappa }$$

Equations (6) and (7) indicate that the central displacement change ratio in regard to the bend angle, *A(κ)*, is a function of the design parameters (helical coil diameter, *d*, and coil wire diameter, *b*) and the bend curvature, *κ*. Here, there is a possibility that the bend curvature could change during sensor operation and affect the measurement of the bend angle.

Equation (7) can be non-dimensionalized by defining the normalized length change, Λ (length change, ∆*l*, divided by the radius of the coil, *d*/2) and the normalized curvature, Κ (curvature, *κ* multiplied by the coil wire diameter, *b*):

(8) $$\Lambda =\frac{\sqrt{2\left\{1-\text{cos}\left(Κ\right)\right\}}}{Κ}\cdot \phi =Χ\left(Κ\right)\cdot \phi$$

The normalized change rate, Χ(Κ), can be expressed with a Taylor series (see Appendix A for more detail):

(9) $$Χ\left(Κ\right)=\sum_{n=0}^{\infty }\frac{1}{16^{n}}\frac{1}{n!}\frac{1}{{\left(3/2\right)}^{\left(n\right)}}{Κ}^{2n}=1+O{\left(Κ\right)}^{2}\approx 1$$

Equation (9) can be approximated to 1 if the approximation error *O*(Κ)^2^ is small enough.

**Figure 8:**
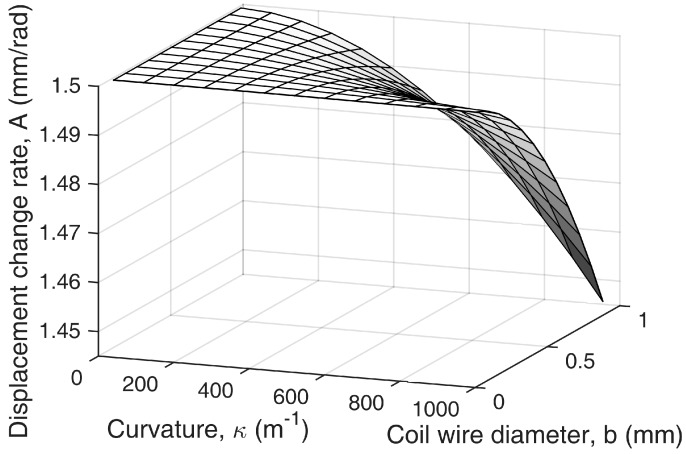
Simulation result showing how the displacement change rate varies with the bend curvature, *κ,* and the coil wire diameter, *b,* when *d* = 3 mm. The displacement change rate can be assumed to be constant within a certain range of design parameters and curvatures.

**Figure 9:**
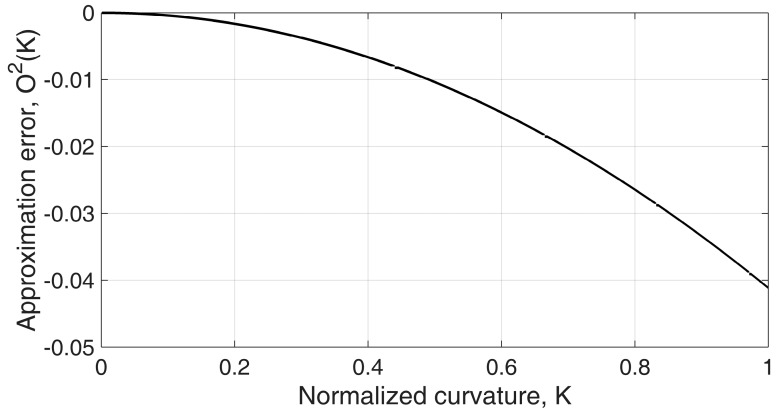
Simulation result of the approximation error depending on the normalized curvature. Large variation of the normalized curvature during sensor operation can lead to measurement error, but not by much—the change rate varies within 1.0% with extreme conditions when Κ = 0.5 (*κ* = 1000 m^−1^ when *b* = 0.50 mm). This can be approximated to a constant value during actual conditions of sensor operation.

Figure 8 and Figure 9 show how the displacement change rate is affected by varying curvature with different design parameters. The displacement change rate is linearly proportional to coil diameter, *d*, as shown in Equation (7), and it has a nonlinear relation to curvature and coil wire diameter, *b*. Figure 8 shows that the displacement change rate, *A*, does not vary much when the coil wire diameter, *b*, is small. This is because the helical coil is approximated to the continuum structure with an incompressible wall inside the curve if the coil wire diameter is small enough relative to the coil diameter. In this case, the displacement change rate can be considered constant regardless of the bend curvature along the sensor. Figure 9 shows that even in an extreme curvature condition, Κ = 0.5 (e.g., *κ* = 1000 m^−1^, *b* = 0.50 mm), the change rate only varies by 1.0%. The ratio of the coil diameter and the wire diameter illustrated here is within the proper range of practical design parameters because a high coil wire diameter increases the stiffness of the sensor, which is not desirable.

The effective displacement change rate, *A_eft_*, which can be used as a sensor gain, can be derived with the approximation in Equation (9):

(10) $$A_{eft}=\frac{d}{2}\cdot Χ\left(Κ\right)\approx \frac{d}{2}$$

Equation (10) indicates that geometric model in Figure 6 can be simply approximated with a perimeter calculation ∆*l* = *d*/2 × *φ* with the small normalized curvature. It can be used for the model-based sensor gain before precise calibration of the bend sensor. The accuracy of the derived model will be compared with experimental results in Section 6.

### 3.3. Advanced Model

The gap between the sensing wire, the Teflon^®^ tube, and the helical coil together with the cross-sectional shape of the coil wire should be considered for a more accurate description of the relationship between the bend angle of the sensor and the central displacement change.

#### 3.3.1. Gap between Parts

**Figure 10:**
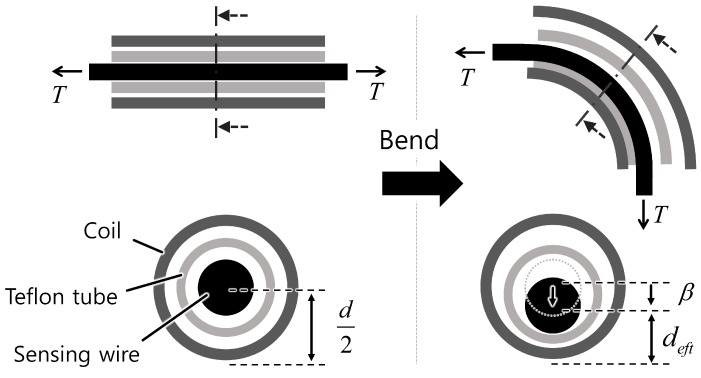
The sensing wire does not maintain its position in the center of the coil because there are gaps between the sensor’s parts. The effective distance between the center of the sensing wire and the outside wall of the helical coil, *d_eft_*, should be considered when calculating the sensor gain derived in Section 3.1.

Gaps between the sensing wire, the Teflon tube, and the helical coil are necessary to prevent excessive friction between these parts and to ensure that the sensing wire slides well with the angle change of the sensor. Thus the sensing wire does not retain its central position inside the coil, and it contacts the inner wall as the helical coil bends when the sensing wire is tensioned. As a result, the effective distance between the sensing wire and the outer wall of the helical coil, *d_eft1_*, is smaller than the radius of the helical coil. This is depicted in Figure 10 and described in the following equation:

(11) $$d_{eft1}=b+b_{tube}+\frac{1}{2}d_{wire}$$

where *b*, *b_tube_*, and *d_wire_* are the wall thickness of the helical coil, the wall thickness of the Teflon tube, and the diameter of the sensing wire, respectively.

#### 3.3.2. Shape of the Cross Section

**Figure 11:**
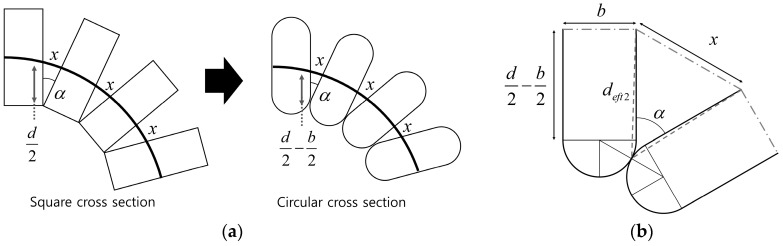
The model should be modified for a coil wire with a circular cross-sectional area for more accurate modeling. (**a**) Model modification for a circular cross section; (**b**) Close view of the contact point between the two coil pitches.

In the helical coil model in Figure 6, the coil wire was assumed to have a square cross-sectional shape for purposes of simplification. However, the actual distance between the contact point of each coil pitch and the sensing wire decreases if the cross section of the coil wire is circular in shape. Figure 11b shows a close up of the contact point between two pitches with rounded ends. The modified distance, *d_eft2_*, can be approximated to a constant value if the angle between two pitches, *α*, is small enough:

(12) $$\underset{\alpha \to 0}{\text{lim}}\;d_{eft2}=\frac{d}{2}-\frac{b}{2}$$

### 3.4. Sensor Gain

The resultant sensor gain, which transforms the measured position of the sensing wire to the bend angle of the sensor, can be derived from the considerations described in Section 3.1, Section 3.2 and Section 3.3:

(13) $$\frac{\phi }{\Delta l}=\frac{1}{\left(\frac{b}{2}+b_{tube}+\frac{d_{wire}}{2}\right)}$$

(14) $$\frac{\phi }{\Delta \theta }=\frac{r}{\left(\frac{b}{2}+b_{tube}+\frac{d_{wire}}{2}\right)}$$

Equations (13) and (14) describe a sensor gain with a linear position transducer and a rotational angle transducer, respectively. The operating range of each position transducer should be determined considering the range of motion of the bend sensor to increase the resolution and the sensitivity of the bend sensor. A small-diameter spool increases the ratio of spool rotation per sensor bend, which will increase resolution and sensitivity but decrease the sensing range.

### 3.5. Axial Force Robustness

**Figure 12:**
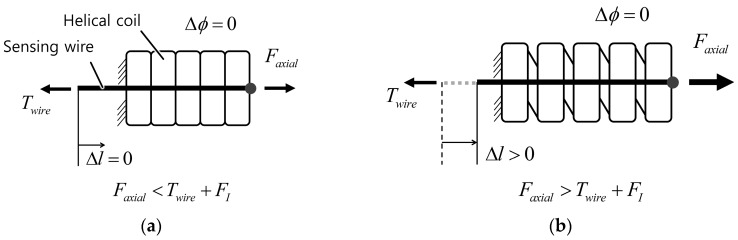
Axial force robustness. (**a**) Safe region; (**b**) A sensing error occurs when the external axial force is higher than the wire tension and the initial tension.

The helical coil can stretch, causing a sensing error, if an axial force is exerted on the sensor, as shown in Figure 12. Stretching of the helical coil along the axial axis is prevented by the tension of the sensing wire and the initial extension force of the helical coil. However, when the external axial force is higher than the summation of the wire tension and the initial tension, a sensing error results for the bend sensor. The robustness of the axial force can be increased by choosing a helical coil with a higher initial tension (generally proportional to wire diameter and stiffness) and by increasing the pre-tension of the sensing wire.

### 3.6. Axial Rotation Robustness

**Figure 13:**
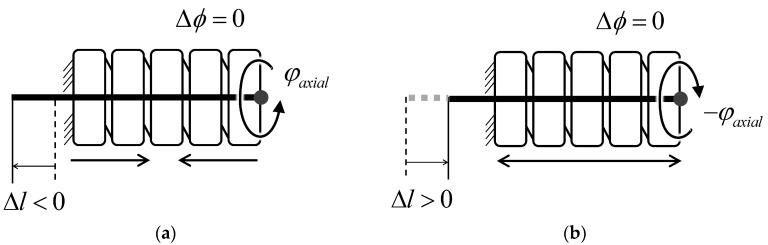
Axial rotation along the helical coil causes only a small length change of the helical coil. The sheath (**a**) contracts with counter-clockwise rotation and; (**b**) extends with clockwise rotation.

Axial rotation of the helical coil can cause geometrical changes in the sheath. The counter-clockwise rotation shown in Figure 13a further coils the sheath, which increases the total length of the sheath, as if the sensing wire were being pulled by the sensor’s bending:

(15) $$\Delta L=\frac{b}{2\pi }\varphi_{axial}$$

The length change of the sheath can be represented as shown in Equation (15), where *ϕ_axial_* is the axial rotation angle of the sheath along the sensor in the radian. As an example, 90° rotation of the sheath clockwise will increase sheath length 0.125 mm, causing a measurement error of 4.9°. However, this effect is much smaller in the real world because there is friction between each coil pitch and the diameter of the helical coil does not change uniformly along the sheath.

## 4. Characteristics of the Proposed Bend Sensor

**Figure 14:**
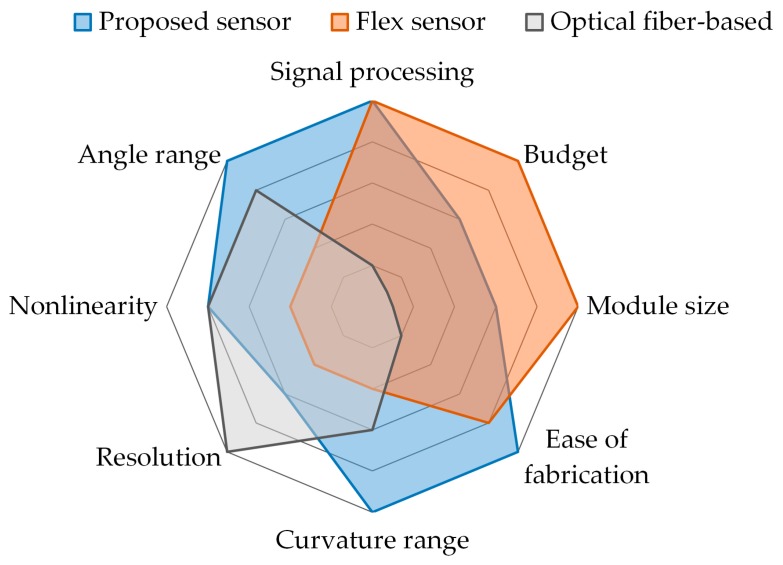
Comparison of the representative characteristics between the proposed sensor, the flex sensor, and the optical fiber-based sensor.

The proposed sensor has several characteristics that differentiate it from flex sensors and optical-fiber based sensors. The properties of these sensors are compared in Table 1 and Figure 14.

1. Low cost
  The proposed sensor is made of low-cost materials and can be manufactured easily without specialized fabrication techniques. The sensor can be assembled with off-the-shelf parts within 15 min.
2. High degrees of freedom for the sensor’s dimensions
  The dimensions of the sensor are determined by the diameter and length of the helical coil. Thus the size of the sensor can be easily and finely adjusted because there is a wide range of choices for the diameter of the extension spring, and the spring can be cut to the desired size.
3. Intuitive and easy to use
  The principle of the proposed sensor is the mechanical conversion of information about bending to information about displacement of the sensing wire. Thus the measurand of the proposed sensor is the displacement of the sensing wire. Because there are various options for the displacement transducer, the user can select one based on their preferences. No complex signal processing or specialized data acquisition device is required because the measurement from the displacement transducer is proportional to the bend angle of the sensor.
4. No directionality
  This sensor can detect bending in any direction because the helical coil’s shape and properties are symmetrical along the axial line. And because the sensing wire is placed in the center of the helical coil, the axial displacement of the sensing wire changes with bending in any direction along the helical coil.
5. Measures the accumulated bend angle
  This sensor cannot detect the direction of bending or the specific location where bending occurs because it only measures one-degree-of-freedom information, that is, central displacement changes of the coil. However, it can detect the accumulated bend angle along the sensor, as depicted in Figure 4, because any local bending along the helical coil causes a central displacement change along the sheath.
6. Large sensing range
  The sensor’s physical sensing range is restricted only by the maximum bend curvature of the helical coil that does not cause permanent strain on the sheath. Widely used extension springs made of spring steel or stainless steel have high elasticity and can enable large curvature of the sensor.
7. Theoretical performance
  The theoretical performance of the sensor depends on the design parameters and performance of the sensor that is used to measure the displacement change of the sensing wire. The Hall-effect sensor provides high performance for both a linear and a rotary configuration. An incremental Hall-effect encoder with 8192 counts per resolution can provide 0.2° resolution for a bend sensor consisting of a 3-mm outer diameter helical coil and a 6-mm diameter spool. A potentiometer is an economical option that provides sufficient performance.
8. Bend stiffness
  The bend stiffness of the sensor is determined by the stiffness of the helical coil and the tension of the sensing wire. A large spring constant and a large-diameter coil lead to high bend stiffness of the sensor because energy is required to elongate the spring coil, as shown in Figure 6. Also, greater tension in the sensing wire and a larger helical coil diameter contribute to the restoring force from the sensing wire, which increases the bend stiffness. Bend stiffness increases as the sensor bends if a spring is used to provide tension on the sensing wire. Because bend stiffness can be modeled as a function of design parameters, a bend stiffness profile can be adjusted by changing the radius of the spool and the constant of the linear spring. An actuator can be used as a tensioning device to provide more degrees of freedom for the stiffness profile or to allow variable sensor stiffness.
9. Environmental robustness
  The absence of electronic components on the bending part ensures good robustness to environmental conditions. Stainless steel, Teflon, and aramid, which are used in the helical coil, liner, and sensing wires, respectively, have high chemical resistance and thermal resistance (the melting point of Teflon and aramid is 327 °C and 500 °C, respectively). Because the sensor is mechanically operated, it is immune to electromagnetic interference and moisture, allowing it to be used even when submerged in boiling water or high-temperature oil.

## 5. Prototype Development

**Figure 15:**
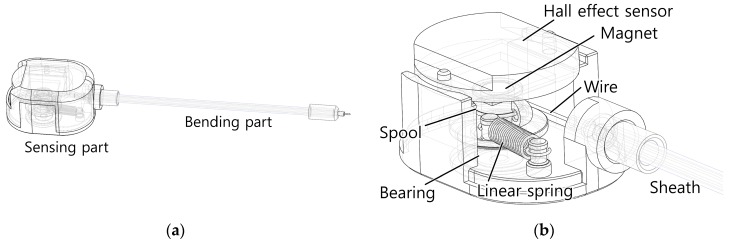
Computer-assisted design (CAD) model of the bend sensor prototype. (**a**) Overall view of the sensor consisting of a sensing part and a bending part; (**b**) Close view of the sensing part, which provides tension to the sensing wire and measures its position.

**Figure 16:**
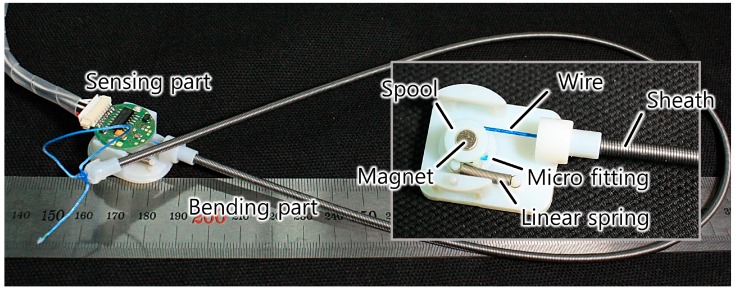
Developed prototype of the bend sensor.

A prototype of the proposed bend sensor was developed using a spool to provide internal tension and measure the position change of the sensing wire (Figure 15 and Figure 16). A miniature extension spring was attached between the spool and the frame to provide a restoring torque to the spool. A Hall-effect angle sensor and a magnet were placed to measure the rotational angle change of the spool. The Hall-effect sensor provides higher resolution than a rotary potentiometer, but it is more expensive. The frame of the sensing part was fabricated using a three-dimensional (3D) printer. A continuous length extension spring (Φ3.0 mm × Φ2.0 mm × 500 mm—Φ indicates diameter) whose coil wire had a circular cross section was used as the helical coil of the bending part. An extension spring can be cut to the desired length of the sensor, and it is easy to find an off-the-shelf continuous length spring up to 1 m long on the market.

A Teflon tube was used inside the coil to reduce friction along the sensing wire. Two Teflon tubes with different diameters (Φ1.38 mm × Φ1.88 mm and Φ1.40 mm × Φ0.90 mm) were fitted together to increase the wall thickness and place the sensing wire as close as possible to the center of the coil, which increases the ratio of the central displacement change to bending of the sensor. The sensing wire and Teflon tube should not be tightly fit because that causes too much friction, resulting in hysteresis of the sensor. Using a 0.9 mm inner diameter Teflon tube with a 0.45 mm diameter Dyneema (Royal DSM, Heerlen, Netherlands) wire ensures that the wire moves smoothly. A 3D-printed end-cap was attached to both ends of the helical coil to provide rigidity and to connect the coil to the frame and fix the end of the sensing wire. The sensing wire was built with braided Dyneema wire (Φ0.45 mm) because this material has high modulus (109–132 GPa tensile modulus [37]), ensuring that the wire did not elongate and violate the assumptions of the derived model. Dyneema wire is flexible and easily wound around a small-diameter spool (6 mm), and it does not lose contact with the spool and the inner wall of the Teflon tube. Metal wires can lose contact with the surface of a spool because their bending stiffness is higher than that of polymer wires. Also, Dyneema wire is reported to have a small friction coefficient when used with Teflon tubes (a friction coefficient of 0.055 compared to 0.092 for steel [36,38]).

All of the materials used to fabricate the sensor are inexpensive, enabling manufacture of a low-cost bend sensor. The total price of the components used, exclusive of the displacement sensing element, was $2.60 US dollars. This study used a Hall-effect sensor module with an embedded analog to digital converter (ADC), which is somewhat expensive for fast prototyping. Using Hall-effect sensor ICs with their own electronics would further reduce the manufacturing cost. The total cost of the sensor could be reduced to $3.60 US dollars if a rotary potentiometer is used instead of a Hall-effect sensor. The specifications and prices of the components are listed in Table 2.

## 6. Experiments

Two experiments were conducted to determine the output response of the sensor in terms of the reference bend angle and curvature. First, a quasi-static experiment was conducted to compare the continuous response of the sensor at a bend angle from 0° to 120° using an external reference angle sensor. Limitation of the bend angle range in the quasi-static experimental setup encouraged a second experiment that permitted large deflections. Finally, a static experiment was conducted to determine the response of the sensor to large deflections (0° to 450° bend angle) with different bend curvatures (*κ* = 26.7 m^−1^, 40.0 m^−1^, and 80.0 m^−1^) using bending brackets.

Hall-effect sensors with an incremental quadrature pulse output (RMB20IC; RLS, Komenda, Slovenia, accuracy ±0.7°, hysteresis 0.18°) and a magnet (Φ4 mm × 4 mm) were used to measure the spool angle of the bend sensor and the reference angle. The output signal of the sensor and the reference angle were acquired with a data acquisition device (cRIO-9082, National Instruments Corp.) with a differential digital input module (NI 9411, National Instruments Corp.). Data was saved on a host PC using an Ethernet interface with a 1-kHz sampling frequency. No filter was applied after data acquisition because the Hall-effect sensor module included an embedded ADC with filters. All of the brackets used in both experiments were made with a 3D printer (Object Connex, Stratasys Ltd.).

### 6.1. Quasi-Static Experiment

**Figure 17:**
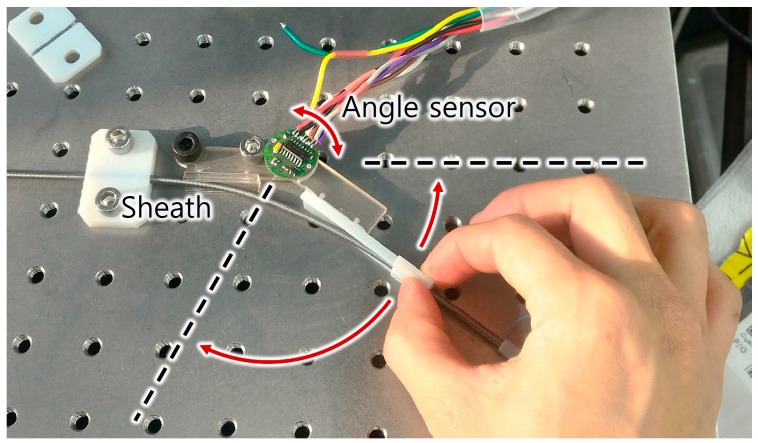
Setup for the quasi-static experiment. The sensor sheath was constrained to the desired bend angle, and the setup was moved by hand.

Figure 17 shows the experimental setup for measuring the sensor signal with a continuous bend angle change from 0° to 120°. To set the desired rotation angle, a rotating bracket with a reference angle sensor was moved by hand. The moving bracket was rotated from 0° to 120° and 120° to 0° three times sequentially.

**Figure 18:**
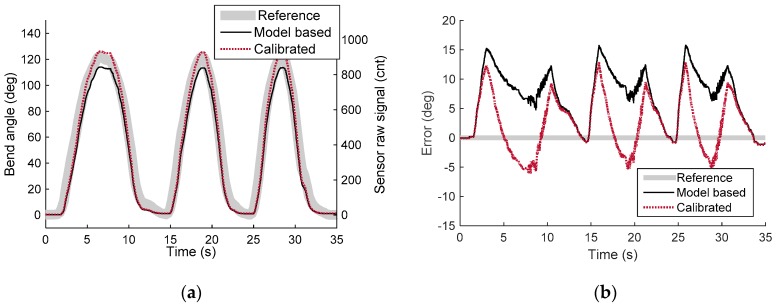
Results for the quasi-static experiment in the time domain. (**a**) Reference angle (gray thick line), model-based sensor output (black solid line), and calibrated sensor output (red dotted line) in the time domain; (**b**) Sensing error of model-based sensor output (black solid line) and calibrated sensor output (red dotted line) in the time domain.

**Figure 19:**
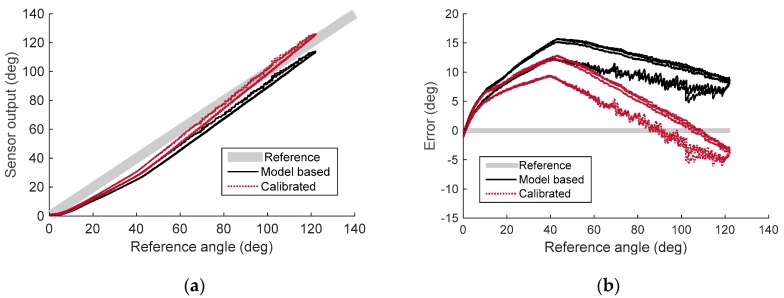
Results for the quasi-static experiment in the reference angle domain. (**a**) Reference angle (gray thick line), model-based sensor output (black solid line), and calibrated sensor output (red dotted line) in the reference angle domain; (**b**) Sensing error of model-based sensor output (black solid line) and calibrated sensor output (red dotted line).

Figure 18 and Figure 19 show the experimental results of the quasi-static experiment in the time domain and the reference angle domain, respectively. Data was plotted to compare the reference angle, the model-based sensor output, and the calibrated sensor output. The model-based sensor output was calculated from the derived sensor model, the design parameter of the prototype, and the raw signal from the Hall-effect sensor. It shows the modeling accuracy of the sensor model derived in Section 3. The result shows that the model-based sensor output was lower than the actual reference angle, with a root mean square error (RMSE) of 8.42°. This means that the actual displacement change of the sensing wire is lower than for the derived model because of the uncertainty of the model. However, Figure 19a shows that the sensor output signal has high linearity and can be calibrated with a linear function to increase accuracy. Linear regression (ordinary least squares) without the intercept term was conducted on the raw signal of the sensor and the reference angle to calibrate the developed sensor. The calibrated signal was plotted with a red dotted line in Figure 18 and Figure 19. The RMSE of the calibrated signal was reduced to 5.15°, which is 4.29% of the full scale (120°). The R-square value (coefficient of determination) of the calibrated sensor output is 0.9887, which ensures the sensor to be used with linear calibration function. However, the sensor signal can be calibrated with polynomials to further increase sensing accuracy. 

The results show low levels of hysteresis with a maximum offset of about 3°. The sensor’s hysteresis may have originated in wire friction. It can be compensated for with hysteresis modeling [32,33,34,35] or minimized by hysteresis analysis and optimization of the design parameter of the sensor.

### 6.2. Static Large Deflection Experiment

**Figure 20:**
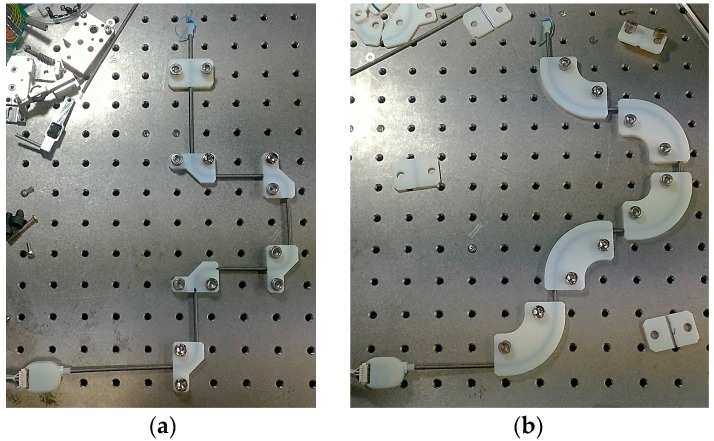
Setup for the static large deflection experiment using a 90° bending bracket with three different curvatures. (**a**) *κ* = 80.0 m^−1^; (**b**) *κ* = 26.7 m^−1^.

A static large deflection experiment was conducted to determine the sensor’s response when subjected to large bend angles of different curvatures. Five brackets having a 90° bend angle were placed onto breadboard for experiments with three different bend curvatures (*κ* = 26.7 m^−1^, 40.0 m^−1^, and 80.0 m^−1^), as shown in Figure 20. Sensor output was measured with 11 different bend angles by adding and subtracting brackets from 0° to 450° and 450° to 0°.

**Figure 21:**
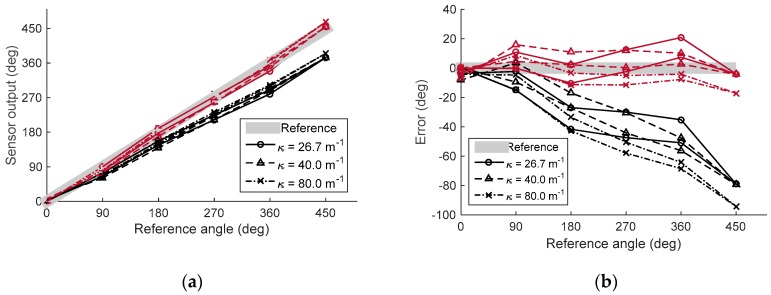
Experimental results showing the output signal with various bending angles and curvatures. (**a**) Sensor output with difference curvatures showing model-based output (black) and calibrated output (red); (**b**) Sensing error with different curvatures, showing model-based output (black) and calibrated output (red).

Figure 21 shows the result of the static large deflection experiment. Data was plotted with different curvatures, and the sensor signal was compared to the reference angle. Overall, it was found that the sensor output shows a linear increment with an increasing bend angle up to 450°, with R-square values of 0.9959, 0.9968, and 0.9969 for the calibrated sensor output for the three different curvatures. Also, the tendency of the sensor output was not affected by the bend curvature, which was the expected result from the modeling and simulation.

The sensor output based on the model is still lower than the reference angle as in the quasi-static experiment, with RMSEs for the model-based sensor output of 38.05°, 37.90°, and 49.39° for the three curvatures. Additionally, the RMSEs of the calibrated sensor output are 9.05°, 8.10°, and 8.29° for the three curvatures, which represents a 2% maximum error of the full sensing range. The sensing range of this experiment was restricted by the length of the sensor. A longer bend sensor would have allowed a larger bend angle to be measured.

## 7. Discussion

The experimental results in Section 7 show some unmodeled measurement error. The expected error of the proposed sensor can be considered in terms of thermal properties and mechanical precision. The helical stainless steel coil had a positive thermal expansion coefficient (α_ss_ = 10.1 × 10^−6^ − 17.3 × 10^−6^ K^−1^), while Dyneema wire had a negative coefficient (α_dyneema_ = −12 × 10^−6^ K^−1^) [37]. This resulted in a 0.011–0.015 mm displacement error and a 0.65–0.86° measurement error per unit temperature (K^−1^) for the 500 mm length of the sensor. However, the huge temperature change that occurred during sensor measurement is not typical, and the temperature effect described above is much smaller than the temperature effect of flex sensors (±30% resistance tolerance) [1]. The precision of parts and the assembly process is another factor affecting the measurement error because the principle of the sensor is the mechanical conversion of the bend angle to the displacement change. Improper finishing of the sensing wire can lead to a dead signal zone or a zero offset change of the sensor. Also, the tilt axis of the spool can affect sensor gain depending on the bend angle, as shown in the tendency of an error in Figure 19. In addition, friction between the sensing wire and Teflon tube can affect the nonlinearities of the sensor signal, although the tension of the sensing wire is kept as low as possible to reduce the friction effect. Furthermore, during long-term use of the sensor, sensor contamination can affect its friction and hysteresis characteristics.

## 8. Conclusions

This study proposes a new type of bend sensor that uses a Bowden-cable and a displacement sensing element. The basic principle of the sensor is based on the displacement change of the inner wire when the shape of the sheath changes, which previous studies have considered to be an error. It has been shown that the output signal of the sensor has high linearity with the bend angle and can measure a wide range of accumulated bending angles with large curvatures.

The proposed bend sensor has three main advantages over previous bend sensors, including flex sensors and optical fiber-based sensors. First, it is low cost. The design and manufacturing process for the proposed sensor are very simple. It can be easily made in laboratories with cheap and affordable parts that do not require any special fabrication technique. Second, it is easy to use. It does not need complicated signal processing because the bend angle of the sensor is proportional to the output signal of the displacement sensing module. Third, it can measure a wide range of bend angles and curvatures (up to 450° bend angle and 80.0 m^−1^ curvature for the first prototype). The sensor signal is linear with large curvatures (R-square value up to 0.9969 with a curvature 80.0 m^−1^), and the length of the sensor can be easily extended to measure large angles.

Nevertheless, this study has some limitations. Some non-linearity, which is shown in the initial bend of the sensor, can accumulate if the sensor is subjected to multiple bend points. However, many applications that use other types of bend sensors do not involve multiple bend points. Also, some sensor nonlinearities can be compensated for with more precise modeling and calibration, because the signal is repeatable.

Despite these limitations, the proposed sensor is expected to overcome the usability and accessibility deficiencies of flex sensors and optical fiber-based sensors. Many existing studies can be further developed by using the proposed bend sensor for more precise control of a Bowden-cable [28,29,30,31,32,33,34,35,36] or to obtain shape feedback from soft-bodied structures like soft robots [39,40].

## Figures and Tables

**Table 1:** Comparison of sensor characteristics.

Property	Proposed Sensor	Flex Sensor	Optical Fiber-Based Sensor
Size	Thin	Wide	Very thin
Length	Unlimited ^1^	Fixed	Unlimited ^1^
Measurand	Position	Electrical resistance	Light ^2^
Module size ^3^	Medium	Compact	Large
Signal processing	Simple	Simple	Complicated
Fabrication	Simple	Off the shelf	Complicated
Cost ^4^	Cheap	Cheap	Expensive
Directionality	Any-direction	Uni-/bi-direction	Any-direction
Curvature range	Wide	Narrow	Restricted
Stiffness ^5^	Adjustable	Fixed	Depends on fiber
Nonlinearity	Low	High	High
Resolution ^6^	High	Low	High

^1^ Theoretically, the length can be increased infinitely, but unmodeled properties can affect the characteristics of sensors if they are lengthened a great deal; ^2^ Measures intensity, phase, wavelength, and polarization of light. An interrogator is necessary for spectrum analysis; ^3^ Size of the device that detects the measurand. It can be a Hall-effect sensor or a potentiometer (as in the proposed bend sensor) or a photodiode (as in an optical fiber-based sensor); ^4^ Costs for the proposed sensor in US dollars: $3.60 for the potentiometer version and $52.60 for the Hall-effect sensor version. Flex sensor: approximately $10 depending on size. Optical fiber-based sensor: Approximately $12,000, the manufacturer's suggested retail price for the interrogator (PXIe-4844; National Instruments, Corp., Austin, TX, USA); ^5^ Bending stiffness of the optical fiber depends on the material and the inner and outer diameter of the fiber; ^6^ Resolution of the proposed sensor depends on the performance of the position measurement of the sensing wire and is bounded by mechanical properties (friction and backlash). On the other hand, the resolution of the optical fiber-based sensor is closely related to its fabrication process and sophisticated signal processing.

**Table 2:** Specification and price list of the prototype components (US dollars).

Component	Specification	Price
Helical coil	Extension spring: Φ3.0 mm × Φ2.0 mm × 500 mm	$1
Lining	Outer Teflon tube: Φ1.38 mm × Φ1.88 mm; Inner Teflon tube: Φ1.40 mm × Φ0.90 mm	$0.2
Wire	Braided Dyneema wire: Φ0.45 mm	$0.1
Structure	3D printed with VeroWhitePlus™ (Objet Connex; Stratasys Ltd., Eden Prairie, MN, USA)	$1
Spring	Miniature extension spring	$0.3
Displacement sensor	Hall-effect sensor: RMB20IC13BC10 (RLS; 8192 pulses per turn, ±0.5° accuracy, 0.18° hysteresis, onboard RC filter with 720 Hz cut-off frequency for sine and cosine signal of magnetic flux); Magnet: Φ4 mm × 4 mm	$50

## Appendix A

The Taylor series for Equation (8) can be derived as follows:

(A1) $$Χ\left(Κ\right)=\frac{\sqrt{2\left\{1-\text{cos}\left(Κ\right)\right\}}}{Κ}=\sum_{n=0}^{\infty }\frac{{Χ}^{\left(n\right)}\left(x_{0}\right)}{n!}{\left(x-x_{0}\right)}^{n}$$

(A2) $${Χ}^{0}\left(0\right)=\underset{Κ\to 0}{\lim}\frac{\sqrt{2\left(1-\text{cos}\;Κ\right)}}{Κ}=1$$

(A3) $${Χ}^{1}\left(0\right)={\left[-\frac{\sqrt{2\left(1-\text{cos}\;Κ\right)}}{{Κ}^{2}}+\frac{\text{sin}\;Κ}{Κ\sqrt{2\left(1-\text{cos}\;Κ\right)}}\right]}_{Κ=0}=0$$

(A4) $${Χ}^{2}\left(0\right)={\left[\frac{2\sqrt{2\left(1-\text{cos}\;Κ\right)}}{{Κ}^{3}}+\frac{\text{cos}\;Κ}{Κ\sqrt{2\left(1-\text{cos}\;Κ\right)}}-\frac{\sqrt{2}\text{sin}\;Κ}{{Κ}^{2}\sqrt{1-\text{cos}\;Κ}}-\frac{{\text{sin}}^{2}Κ}{2\sqrt{2}{\left(1-\text{cos}\;Κ\right)}^{3/2}}\right]}_{Κ=0}=\frac{1}{12}$$

(A5) $$Χ\left(Κ\right)=1-\frac{{Κ}^{2}}{24}+\frac{{Κ}^{4}}{1920}-\frac{{Κ}^{6}}{322560}+\frac{{Κ}^{8}}{92897280}+\cdots$$

(A6) $$Χ\left(Κ\right)=\sum_{n=0}^{\infty }\frac{1}{16^{n}}\frac{1}{n!}\frac{1}{{\left(3/2\right)}^{\left(n\right)}}{Κ}^{2n}$$

where *x^(n)^* is the Pochhammer symbol [41],

(A7) $$x^{\left(n\right)}\equiv \frac{\Gamma \left(x+n\right)}{\Gamma \left(x\right)}=x\left(x+1\right)\cdots \left(x+n-1\right)$$
